# Neuroprotective Effects of Endogenous Secretory Receptor for Advanced Glycation End-products in Brain Ischemia

**DOI:** 10.14336/AD.2019.0715

**Published:** 2019-07-15

**Authors:** Yu Shimizu, Ai Harashima, Seiichi Munesue, Masahiro Oishi, Tsuyoshi Hattori, Osamu Hori, Yasuko Kitao, Hiroshi Yamamoto, Nontaphat Leerach, Mitsutoshi Nakada, Yasuhiko Yamamoto, Yasuhiko Hayashi

**Affiliations:** ^1^Department of Biochemistry and Molecular Vascular Biology,; ^2^Department of Neurosurgery and; ^3^Department of Neuroanatomy, Kanazawa University Graduate School of Medical Sciences, Kanazawa 920-8641, Japan.; ^4^Komatsu University, Komatsu, Ishikawa 923-8511, Japan.; ^5^Department of Neurosurgery, Kanazawa Medical University, Uchinada 920-0293, Japan.

**Keywords:** Receptor for advanced glycation end-products (RAGE), endogenous secretory RAGE (esRAGE), parabiosis, delayed neuronal cell damage, blood-brain barrier

## Abstract

The receptor for advanced glycation end-products (RAGE) is expressed on human brain endothelial cells (HBEC) and is implicated in neuronal cell death after ischemia. We report that endogenous secretory RAGE (esRAGE) is a splicing variant form of RAGE that functions as a decoy against ischemia-induced neuronal cell damage. This study demonstrated that esRAGE was associated with heparan sulphate proteoglycans on HBEC. The parabiotic experiments between human esRAGE overexpressing transgenic (Tg), RAGE knockout (KO), and wild-type (WT) mice revealed a significant neuronal cell damage in the CA1 region of the WT side of parabiotic WT→WT mice, but not of Tg→WT mice, 7 days after bilateral common carotid artery occlusion. Human esRAGE was detected around the CA1 neurons in the WT side of the parabiotic Tg→WT pair, but not in the KO side of the Tg→KO pair. To elucidate the dynamic transfer of esRAGE into the brain, we used the blood-brain barrier (BBB) system (PharmaCo-Cell) with or without RAGE knockdown in endothelial cells. A RAGE-dependent transfer of esRAGE was demonstrated from the vascular to the brain side. These findings suggested that esRAGE is associated with heparan sulphate proteoglycans and is transferred into the brain via BBB to exert its neuroprotective effects in ischemia.

Cerebral ischemic injury is one of the main causes of death and disability. Ischemic stroke, which results in insufficient supply of glucose and oxygen to brain tissues, could cause significant neuronal damage leading to neuronal cell death [1, 2]. Transient global cerebral ischemia induces delayed neuronal death in the hippocampal CA1 subfield [3, 4]. The hippocampus is not only responsible for many central nervous system functions including cognition, learning, and memory but is also one of the most vulnerable brain regions to various neurological insults such as hypoxia-ischemia, seizure, and prolonged stress [5-8]. The CA1 subfield has low ischemic tolerance, similarly to the striatum, thalamic reticular nucleus, and cerebellar Purkinje cells [9-11]. There are many hypotheses about the molecular mechanisms underlying the delayed neuronal cell death in ischemia, including mitochondrial dysfunction, oxidative stress, nitrosative stress and the calcium-glutamine hypothesis [12-15].

The receptor for advanced glycation end-products (RAGE) is a pattern-recognition receptor that is involved in ischemic brain injury [16-18]. It is also well known that RAGE is implicated in the development of diabetic vascular complications, atherosclerosis, neuro-degenerative diseases, and inflammatory diseases [19-24]. In the brain, RAGE expression is reported in microglial and endothelial cells [12, 25]. Kamide *et al*. demonstrated that the expression of RAGE could rise in the cerebrovascular endothelium and hippocampal neurons after transient brain ischemia by bilateral common carotid artery occlusion (BCCAO) [16]. We have previously identified and reported endogenous soluble RAGE (esRAGE), which is a truncated variant of RAGE isoforms generated by alternative splicing of the pre-mRNA transcripts and detectable in human circulation [26]. Recombinant proteins of the soluble form of RAGE were generated with gene technology and used in animal experiments [27, 28]. Soluble forms of RAGE proteins are known to be generated in vivo by cleavage of membrane-bound full-length RAGE as well as esRAGE by metalloproteinsases [22]. The soluble RAGE proteins including esRAGE and the cleaved forms of RAGE are known to act as a decoy receptor and to ameliorate pathologies in experimental animal models [29]. For example, the application of soluble RAGE significantly reduced the infarct size and showed neuroprotective effects on cortical neurons in mouse MCAO models [30, 31]. In addition, the expression of inflammatory molecules such as tumor necrosis factor α or inducible nitric oxide synthase was attenuated at the mRNA level in the hippocampal CA1 region of esRAGE transgenic (Tg) mice and in RAGE knockout (KO) mice, when compared with wild-type (WT) mice [16]. The neuroprotective role of esRAGE was observed. Regarding the esRAGE Tg mice used in the report, the mice were created to produce human esRAGE in the liver using the albumin promoter [28]. However, human esRAGE was also detected in the brain of this mouse model. Therefore, to evaluate the strict effect of only bloodstream esRAGE, we employed the parabiotic strategy [32], which will demonstrate a stable level of blood esRAGE, as a pilot study of intravenous injection of esRAGE protein.

In the present study, we found that blood esRAGE significantly inhibited transient brain ischemia-induced neuronal cell damage and apoptosis in the hippocampal CA1 subfield in the mouse BCCAO model. Blood esRAGE was transferred into the brain through the blood- brain barrier (BBB) by RAGE on endothelial cells, potentially contributing to the neuroprotective effects in ischemia. Thus, endothelial RAGE has two different roles in ischemia, namely as an inducer of vascular injury and neuronal damage and as a transporter of esRAGE, a neuroprotector, to the brain.

## MATERIAL AND METHODS

### Animals

All animal experiments were conducted according to the Guidelines for Proper Conduct of Animal Experiments by the Science Council of Japan (1 June 2006) (www.scj.go.jp/ja/info/kohyo/pdf/kohyo-20-k16-2e.pdf). All animal procedures were performed in accordance with and approved by the Animal Care and Use Committee of Kanazawa University (AP-132929). The report was written in compliance with the ARRIVE guidelines (Animal Research: Reporting in Vivo Experiments) (www.nc3rs.org.uk/arrive-guidelines). The RAGE KO (*Ager*^-/-^) and WT (C57BL/6J) mice were produced by crossbreeding heterozygous mutant mice [20]. The esRAGE Tg (C57BL/6J) mice were generated as described previously [28]. Female mice of each cohort (age, 8-12 weeks; weight, 20-30 g) were used in the experiments. General anesthesia was induced with 2.0% sevoflurane and was maintained with 0.5% sevoflurane using a face mask [16]. To introduce parabiosis, mirror image incisions were made through the skin at the left and right flanks. The skin of the adjacent parabiont was sutured together. Before surgery and 14 d post-operatively, venous blood samples (100 µl) were collected from the tail vein. esRAGE levels in sera, culture media and tissue lysates were measured by human esRAGE enzyme-linked immunosorbent assay (ELISA) kit (B-Bridge) [28]. Cross-circulation was confirmed in a subset of parabiotic pairs by measuring the esRAGE blood concentration from one partner in the blood of the other partner [32]. Serum mouse soluble RAGE (sRAGE) was assayed with a mouse RAGE Quantikine ELISA kit (MRG00, R&D Systems, Inc., MN, USA). Protein concentrations of tissue lysate were measured using a BCA protein assay kit (Pierce Biotechnology, Rockford, IL, USA).

### Surgical procedures (BCCAO)

Seven days after parabiosis, brain ischemia was induced by BCCAO for 20 min with the use of microvascular clips as described previously [16]. Laser-Doppler flowmetry was used to measure the cerebral cortical microperfusion (3 mm lateral to bregma). In our experimental model, mice that demonstrated < 15% of baseline control microperfusion during the first minute of occlusion were used for subsequent experiments. Rectal temperature was maintained at 36.5-37.5 °C by using a heat lamp and a blanket [16].

### Cell culture and immunofluorescence study

Human microvascular endothelial cells (HMVEC) isolated from the neonatal dermis were maintained in HuMedia-EB2 medium supplemented with Humedia-MvG (KURABO KE6550) containing 5% (v/v) fetal bovine serum (FBS), 5 ng/ml basic fibroblast growth factor (bFGF), 10 μg/ml heparin, 10 ng/ml epidermal growth factor (EGF), 1 μg/ml hydrocortisone, and 39.3 μg/ml dibutyryl cAMP, according to the manufacturer’s instructions (Kurabo). Primary human brain microvascular endothelial cells (HBEC) were cultured with the CSC complete Recombinant Medium (Cell Systems Corporation) and cultivated in a humidified 37 °C incubator with 95% room air and 5% CO2. Experiments were performed in an 8-well chamber slide (LAB-TEK™ CHAMBER SLIDE™ SYSTEM, 177445JP). Recombinant human esRAGE protein produced previously in our lab was used for the experiments [26, 28, 33]. Briefly, esRAGE (1 µg/ml) was added to the culture media and incubated for 1 h with or without treatment with heparin (0.1 IU/ml) or heparitinase (1 mU/ml). After washing, cells were fixed with 4% paraformaldehyde for 30 min, and the esRAGE signal was detected with the primary polyclonal anti-esRAGE antibody (1:200) in combination with the secondary antibody Alexa 488-conjugated goat anti-rabbit (Alexa Fluor® 488 Goat Anti-rabbit IgG [H+L]; 1:500; Molecular Probes®) [25, 33, 34, 35]. For the detection of heparin sulfate, we used a monoclonal anti-heparan sulfate (10E4) antibody (Seikagaku Corporation, Japan) in combination with Alexa 647-comjugated goat anti-mouse IgM antibody (ab150123, Abcam). Coverslips were mounted onto glass slides using mounting medium (UltraCruz™ Mounting Medium, Santa Cruz Biotechnology) [28]. Images were acquired by using wide-field fluorescence microscopes (EVOS® FL, Advanced Microscopy Group or BZ-9000, Keyence Co.)

### Immunohistochemistry 

Following transcardial perfusion with 4% para-formaldehyde, brains were dissected and post-fixed in 4% paraformaldehyde for 24 h at 4 °C. Coronal sections (thickness 5 μm) of paraffin-embedded specimens were cut on a cryostat. Deparaffinization was performed by incubating the slides in the following solutions: (1) xylene bath (twice for 5 min each), (2) 100% ethanol (twice for 3 min each), (3) 90% ethanol, (4) 80% ethanol, and (5) double distilled water [4]. Subsequently, the slides were heated in 6 mM sodium citrate buffer (pH 6.0) at 95-100 °C for 10 min by using a microwave, and incubated at room temperature for 30 min in phosphate-buffered saline (PBS) and 1% bovine serum albumin (BSA), followed by anti-esRAGE antibody in combination with Alexa 488-conjugated goat anti-rabbit (Alexa Fluor® 488 Goat anti-rabbit IgG [H+L] antibody, 1:500, Molecular Probes®) in PBS 1% BSA [25]. Images were obtained by using a wide-field fluorescence microscope (EVOS® FL, Advanced Microscopy Group). For co-staining for neuronal or astrocyte marker and human esRAGE, mouse brains were removed after perfusion with 4% paraformaldehyde, and coronal sections (20-μm-thick sections) were cut on a cryostat (CM1950, Leica Biosystems, Wetzler, Germany) after cryoprotection using 30% sucrose. Sections were processed for immunostaining with antibodies against NeuN (MAB377, Millipore, Billerica, MA), GFAP (G3893, Sigma, St. Louis, MO) and human esRAGE [26]. Alexa488 (Invitrogen, Carlsbad, CA)- conjugated donkey anti-mouse or Cy3-conjugated anti-rabbit secondary antibody (Jackson ImmunoResearch Laboratories, West Grove, PA) were used for visualization of immunolabeling. Imaging was performed on a laser scanning confocal microscope (Eclipse TE2000U, Nikon, Tokyo, Japan) with the Nikon EZ-C1 software.

### Neuronal cell quantification

Hippocampal cell death was assessed according to a standard protocol described previously [36, 37]. Brain sections were stained with cresyl violet (Nissl) staining and hematoxylin-eosin staining. Cell counting was performed in five sections per hippocampus starting at 1.4 mm posterior to bregma encompassing the core of the lesioned area (rostrocaudal levels, -1.4, -1.5, -1.6, -1.7, and -1.8 mm from bregma). Injury to the CA1 subfield was evaluated in the medial and intermediate portion of the CA1 under a light microscope at a 100× magnification by manually counting [38]. We defined degenerating neurons as cells with pyknotic, shrunken nuclei and reduced size. Surviving neurons (Nissl-positive and no sign of neurodegeneration) were counted in five fields and expressed as the average number. For the detection of neuronal apoptosis, we used the Apop-Tag Fluorescein *in situ* Apoptosis Detection Kit (Chemicon International) according to the manufacturer’s instructions [39]. Apoptotic neurons (caspase 3-positive neurons) were counted in five different sections [40]. Cell counting was done with blind assessments by two evaluators.

### Blood-brain barrier assay

For this assay, we used a commercially available BBB kit (MBT-24H, PharmaCo-Cell Co., Nagasaki, Japan), which was composed of monkey brain capillary endothelial cells, rat brain pericytes, and rat brain astrocytes. The *in vitro* BBB models were established within 4 d of seeding the cells according to the manufacturer’s instructions [41, 42]. Trans-endothelial electrical resistance (TEER), which primarily reflects the ?ux of sodium ions through the cell layers in the culture conditions, was measured with an epithelial-volt-ohm meter and an Endohm-6 chamber electrode (World Precision Instruments, Sarasota, FL, USA) [42, 43]. The TEERs of the coated, cell-free filters were subtracted from the measured TEER values of the models and shown as Ω cm^2^. The apparent permeability constants (Papp) between the luminal and abluminal chambers were calculated based on the distribution ratios [42, 44]. The expression of endothelial RAGE was silenced using RAGE small hairpin RNA (shRNA) and the pSilencer 3.0-H1 vector (Ambion, Austin, TX) with Lipofectamine 2000 (Thermo Fisher Scientific) [42, 45]. The RAGE-knockdown efficiency was >90% with our shRNA system (data not shown).

**Figure 1.**
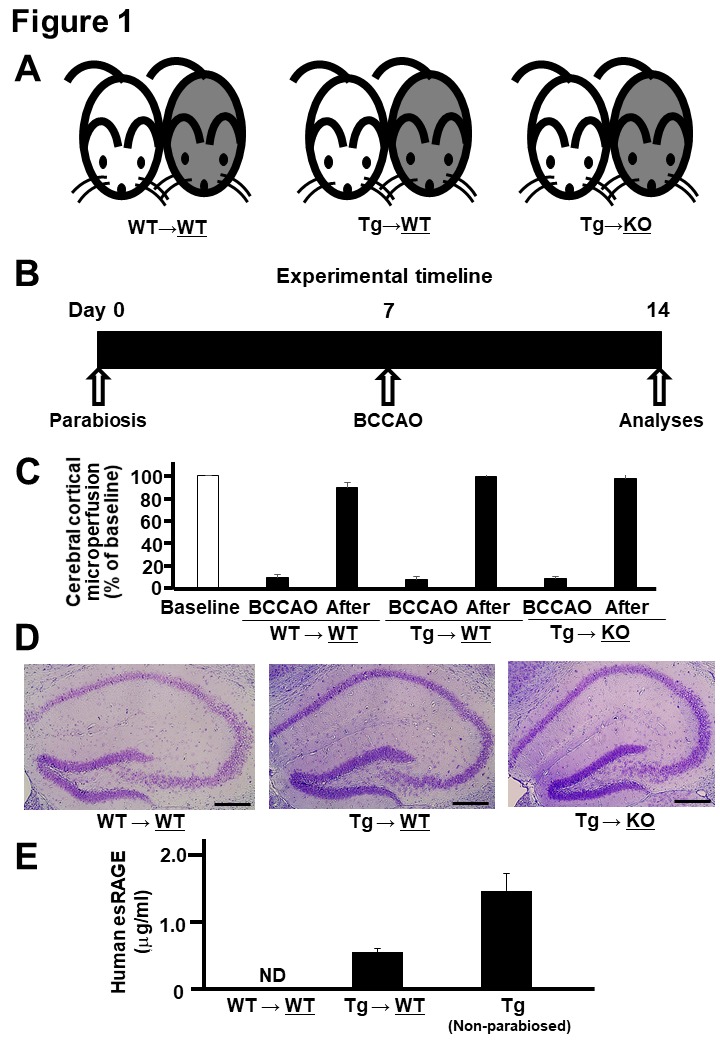
**Parabiosis and BCCAO. A)** Parabiosis was done between wild-type (WT) and WT mice (WT→WT), esRAGE transgenic and WT mice (Tg→WT), and esRAGE transgenic and RAGE knockout (KO) mice (Tg→KO); gray-colored and right-side mice underwent BCCAO and further analyses. **B**) Experimental timeline. **C**, Laser-Doppler flowmetry data for evaluating the cerebral cortical microperfusion. Baseline, baseline data (100%); BCCAO, data at 1 min during the occlusion; After, data at 30 min after BCCAO. Values are mean ± SD. **D**) Hematoxylin-eosin stain of the hippocampus. **E**) Human esRAGE levels in the sera of WT sides of WT→WT and Tg→WT pairs and non-parabiosed esRAGE Tg mice (n = 4-8). ND, not detected. Values are mean ± SEM.

### Statistical analysis

P values were calculated using two-tailed Student’s t-test for pair wise comparisons, and one-way analysis of variance (ANOVA), followed by Bonferroni’s test for multiple comparisons, unless otherwise stated. A P value of < 0.05 was considered statistically significant. These analyses were carried out with the use of Ekuseru-Toukei 2015 (Social Survey Research Information Co., Ltd., Japan).

## RESULTS

### Blood esRAGE protects against neuronal cell damage

To elucidate the functional role of blood circulating esRAGE, we employed mouse parabiosis approaches by using human esRAGE Tg (human esRAGE overexpressed in blood), RAGE KO, and WT mice (Fig. 1A): (1) WT mice parabiosed with WT mice (WT→WT); (2) esRAGE Tg mice parabiosed with WT mice (Tg→WT); (3) esRAGE Tg mice parabiosed with RAGE-KO mice (Tg→KO). By using these three parabiosis pairs, brain ischemia was induced on either side of the pair (gray-colored right one) to investigate the neuronal cell damage and survival 7 d after BCCAO (Fig. 1B). Laser-Doppler flowmetry data showed no differences in ischemic conditions during and after BCCAO among the three groups (Fig. 1C). Severe ischemic brain damages were not observed after BCCAO (Fig. 1D). The transfer of human esRAGE protein from the esRAGE-Tg mice to the blood of WT mice in the Tg→WT pair was confirmed prior to the experiment. The results indicated that the level of human esRAGE in WT mice was the half of that of esRAGE Tg mice in the Tg→WT pair (Fig. 1E).

**Figure 2.**
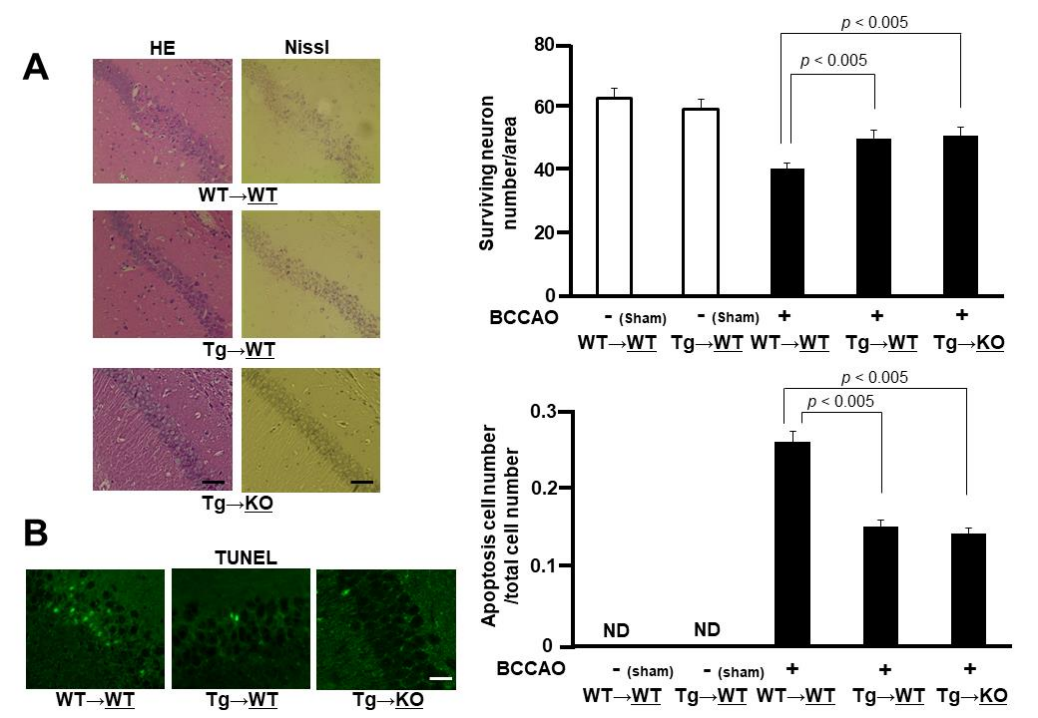
**Neuronal cell damage. A)** HE and Nissl stains of the hippocampal CA1 region of WT sides of WT→WT and Tg→WT pairs and of KO side of Tg→KO pair after 7 days of BCCAO (left panel). Surviving neuron numbers *per* area in hippocampal CA1 region were counted in WT sides of WT→WT and Tg→WT pairs and in KO side of Tg→KO pair with or without BCCAO (right panel) (n = 4-8). Values are mean ± SEM. **B**) TUNEL stain. Green signals indicate apoptotic cells of WT sides of WT→WT and Tg→WT pairs and of KO side of Tg→KO pair after 7 days of BCCAO (left panel). Apoptosis cell numbers *per* total cell numbers were counted in WT sides of WT→WT and Tg→WT pairs and in KO side of Tg→KO pair with or without BCCAO (right panel) (n = 4-8). Values are mean ± SEM.

Subsequently, we investigated the delayed neuronal cell death and cell survival by using a mouse BCCAO model. Both the hematoxylin-eosin and Nissl stainings demonstrated severe neurodegeneration in the CA1 neurons in the WT side of the WT→WT pair, but not in the WT side of the Tg-WT pair, 7 d after BCCAO (Fig. 2A). Quantitative analyses also showed a significant decrease in the number of delayed neuronal cell death in the CA1 region of the WT side of the Tg→WT pair, when compared with that of the WT→WT pair (Fig. 2A). The terminal deoxynucleotidyl transferase dUTP nick end labeling staining was performed to evaluate apoptosis and demonstrated a significantly decreased number of apoptotic cells in the CA1 region of the WT side of the Tg→WT pair, in comparison with the WT→WT pair (Fig. 2B). These results suggested neuroprotective effects of esRAGE. However, we could not find any further synergistic or addictive neuroprotective effects of esRAGE under RAGE-deleted conditions; *e.g.* the KO side of the Tg→KO parabiosis pair (Fig. 2A and B). We speculate that the neuroprotective effects observed in the KO side of the Tg→KO parabiosis pair could be caused by only RAGE-deletion.

**Figure 3.**
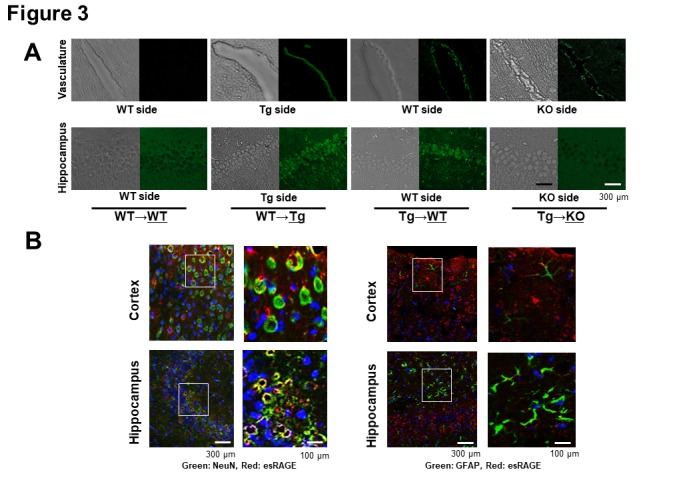
**Immunohistochemical detection of human esRAGE. A)** Immunohistochemical study for the detection of human esRAGE (green signals). Hippocampal vasculatures and CA1 regions of WT side of WT→WT and esRAGE Tg (Tg)→WT pairs, the Tg side of the WT→Tg pair, and the RAGE knockout (KO) side of the Tg→KO pair without BCCAO. **B**) Immunostaining for NeuN (a neuronal marker, green) and human esRAGE (red) (left panel) as well as GFAP (a glial marker, green) and human esRAGE (red) (right panel) in brain cortex and hippocampus of WT sides of Tg→WT pair. Blue signals indicate nuclei [4',6-diamidino-2-phenylindole (DAPI) stain].

### Endothelial RAGE transfers blood circulating esRAGE to the brain

**Figure 4.**
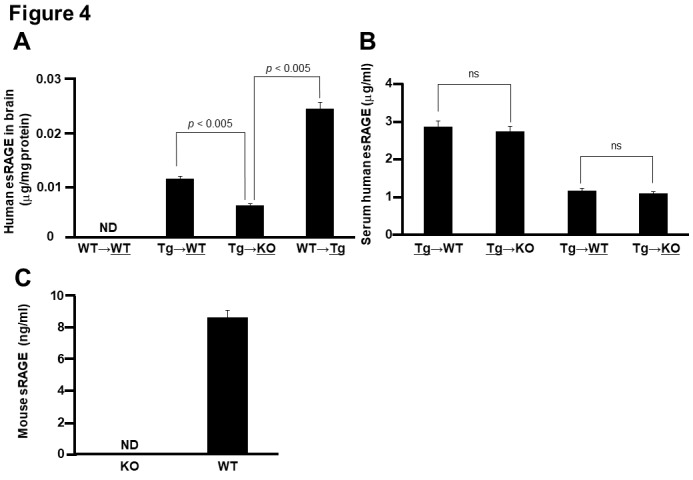
**Quantitative detection of human esRAGE A)** Human esRAGE contents in the brain parenchyma of WT side of WT→WT pair, WT side of Tg→WT pair, KO side of Tg→KO pair, and Tg side of WT→Tg pair (n = 3). **B)** Serum human esRAGE concentrations in Tg side of Tg→WT pair, Tg side of Tg→KO pair, WT side of Tg→WT pair, and KO side of Tg→KO pair (n = 3). **C**, Serum mouse sRAGE concentrations in KO and WT mice (n = 3). ND, not detected; ns, not significant. Values are mean ± SEM.

To address how endothelial esRAGE can protect against neuronal cell death in the ischemic brain, we first checked the *in vivo *presence of the human esRAGE protein by immunofluorescent examinations by using mouse parabiosis models. Therefore, we employed WT→WT, WT→Tg, Tg→WT, and Tg→KO parabiosis pairs and examined the underlined animals in each pair after 7 days of the parabiosis. Based on the results, human esRAGE signals were detected on the brain endothelial surface in the WT→Tg, Tg→WT, and Tg→KO pairs, but not in the WT→WT pair (Fig. 3A). Surprisingly, the human esRAGE protein was also observed in the brain parenchyma around the hippocampal CA1 neurons in the WT→Tg and Tg→WT parabiosis pairs (Fig. 3A). A co-staining experiment also showed that the esRAGE signals were co-localized with NeuN, a neuronal marker, but not GFAP, an astrocytic maker (Fig. 3B). Based on these results, we suggested that the blood circulating esRAGE and endothelium-associated esRAGE proteins were transferred into the brain parenchyma through the BBB. No positive signals of human esRAGE protein were observed in the KO side of Tg→KO or WT→KO pair which was a negative control (Fig. 3A). In addition, a quantitative evaluation with ELISA demonstrated that human esRAGE was detectable and its concentration was significantly lower in the KO side of the brain parenchyma in Tg→KO when compared with the WT side of Tg→WT pair (Fig. 4A). These findings also support our hypothesis that esRAGE transfers into brain through the BBB. The transfer of esRAGE might depend on RAGE, even though some detectable level of human esRAGE was observed in the KO side of Tg→KO pair (Fig. 4A), which may be derived from the contamination of vessel esRAGE. This is based on the fact that serum levels of human esRAGE were 2.0-3.0 µg/ml in the Tg side of Tg→WT and Tg→KO pairs and ~1.0 µg/ml in the WT or KO side of Tg→WT or Tg→KO pairs (Fig. 4B). Mouse sRAGE levels were found to be ~8 ng/ml in WT mice, and at under detectable levels in the KO mice (Fig. 4C), indicating that mouse sRAGE is more than two orders of magnitude lower than human esRAGE levels in the serum of Tg parabiosed mice.

### Association of esRAGE with heparan sulfate proteoglycan (HSPG) of vascular endothelial cells

**Figure 5.**
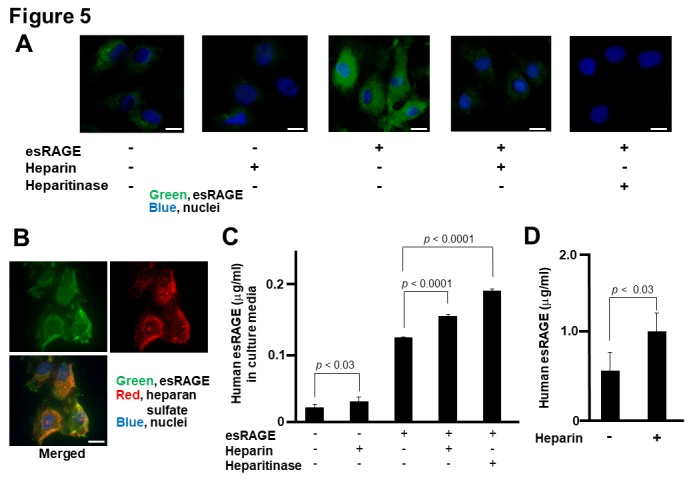
**Association of esRAGE with endothelial cells. A** and **B**) Immunofluorescence studies of human brain endothelial cells (HBEC) in culture. Green, Red and blue signals indicate esRAGE, heparin sulfate and nucleus (DAPI), respectively. Bar, 50 µm. **C**) Human esRAGE levels in culture media of HBEC (n = 4). esRAGE, 1 µg/ml; Heparin, 0.1 IU/ml; heparitinase, 1 mU/ml. Values are mean ± SEM. **C**) Serum levels of human esRAGE in the esRAGE Tg mice with or without heparin injection (n = 4-8). Values are mean ± SEM.

We further investigated whether there was an association between esRAGE and the cell surface of human HBEC in cultures by using fluorescence microscopy. Faint signals of esRAGE were detected in the endothelial cells, which became much more enhanced with the addition of recombinant esRAGE into the cell culture media (Fig. 5A). On the other hand, treatment with heparin (0.1 IU/ml) or heparitinase (1 mU/ml) prevented the accumulation of esRAGE with or without the addition of esRAGE protein in HBEC (Fig. 5A). We obtained the same data in human HMVEC (data not shown). Co-localization of esRAGE and heparin sulfate signals were observed (Fig. 5B). Subsequently, we examined the release of esRAGE protein into the cell culture media of human HBEC with or without treatment with heparin and heparitinase. Based on the results, treatment with heparin (0.1 IU/ml) significantly increased the concentration of esRAGE protein in the cell culture media of HBEC (Fig. 5C). Pretreatment of the HBEC culture media with recombinant esRAGE (1 µg/ml) for 1 h increased the basal level of esRAGE. Similarly, heparin (0.1 IU/ml) or heparitinase (1 mU/ml) also significantly upregulated the esRAGE concentration (Fig. 5C). Furthermore, intravenous heparin injection significantly increased the serum concentration of human esRAGE *in vivo* in the esRAGE Tg mice (Fig. 5D). These results indicate that esRAGE is associated with HSPG of the endothelial cells.

**Figure 6.**
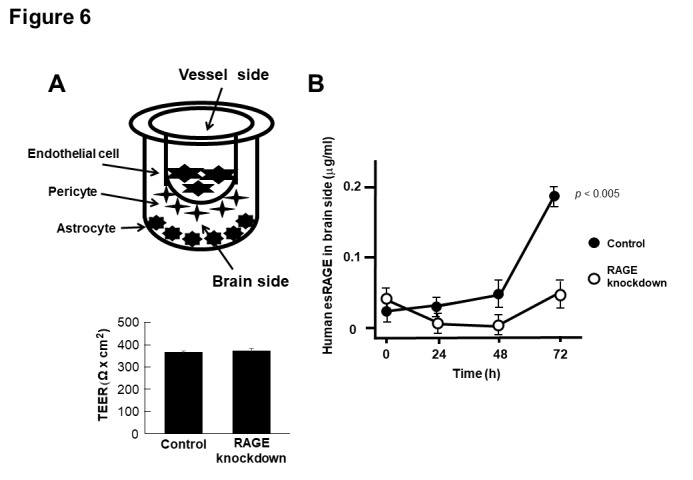
**Transfer of esRAGE through BBB. A**) *In vitro* (BBB) model system composed of primary cultures of monkey brain capillary endothelial cells coupled with rat pericytes and astrocytes. Recombinant esRAGE (20 µg/ml) was added to the upper (vessel side) chambers of the model and transferred esRAGE level was quantified in the lower (brain side) chambers. Endothelial cells were treated with scrambled (control) or RAGE shRNA vectors (knockdown). The integrity of the *in vitro* primate BBB was unaffected by RAGE knockdown, assessed by high trans-endothelial electrical resistance (TEER) (n = 5). **B**) Human esRAGE levels in brain side were quantified (n = 5). Control, scrambled vector-treated; RAGE knockdown, RAGE shRNA vectors-treated. Values are mean ± SEM.

In order to confirm our hypothesis of the esRAGE transfer through the BBB, we used an *in vitro* BBB model composed of brain endothelial cells, pericytes, and astrocytes, whose barrier function was intact with higher TEER levels (Fig. 6A). When 20 μg/ml of human esRAGE protein was added to the vessel side, the esRAGE concentrations of the brain side increased significantly in a time-dependent manner without endothelial RAGE knockdown (control) (Fig. 6B). In contrast, no such increase was observed in the RAGE knockdown model (Fig. 6B), indicating that the transfer of human esRAGE protein occurred through the endothelial RAGE.

### DISCUSSION

Previous reports demonstrated a pivotal role of neuronal and microglia RAGE in ischemia-induced neuronal death and inflammation in mouse models of distal permanent middle cerebral artery occlusion (MCAO), which cause focal ischemic stroke [46, 47]. On the other hand, in the BCCAO model, which is known to induce global ischemia and subsequent delayed neuronal cell death, the induction of RAGE expression in the hippocampal vascular endothelial cells preceded that neurons and glia, indicating that endothelial RAGE could contribute to delayed neuronal death by enhancing vascular injuries, and potentially leading to microcirculatory disturbances [16]. For the therapeutic strategies against stroke and neuronal cell death, both esRAGE and enzymatically cleaved soluble RAGE were applied to ischemic brain injury models of MCAO and BCCAO. Administration of soluble RAGE has been reported to reduce the infarct size of the brain and microglia/macrophage infiltrations in a mouse MCAO model [48]. Using esRAGE-Tg mice, delayed neuronal cell death in the hippocampus was attenuated in the BCCAO model [16]. However, the precise mechanisms of the decoy-type receptor of soluble RAGE and esRAGE in the protection against ischemia-induced neural damage are still unclear.

In this study, we first observed that esRAGE was associated with HSPG of the vascular endothelial cell surface in which it accumulated both *in vitro* and *in vivo* (Fig. 1), which is consistent with previous findings [49]. We also found that excessive circulating esRAGE was protective against delayed neuronal damage in ischemia in our parabiosis models (Fig. 2). Therefore, this study demonstrated for the first time that esRAGE was transferred into the brain from blood through the BBB and localized around the hippocampal CA1 neurons (Fig. 3). These results suggested a functional role of the decoy type receptor against RAGE ligands including damage-associated molecular patterns released in brain ischemia. The human esRAGE is approximately 50 kDa (of a large molecular size) and is usually considered to be untransportable through the BBB. However, using Tg→WT and Tg→KO parabiosis mice in addition to the *in vitro* BBB model with or without RAGE knockdown, we found that endothelial RAGE could function as a transporter of esRAGE protein from blood to brain (Figs. 3-6). We speculated that RAGE may form an oligomer complex with esRAGE on endothelial cells, which has been previously described whereby RAGE itself forms oligomer with its extracellular domain and transcytoses esRAGE from the luminal to the abluminal side [50]. These findings suggested that both circulating and endothelium-associated and accumulated esRAGE could protect against vascular damages in ischemia; similarly, the esRAGE transferred into the brain could also function as a neuroprotective factor in ischemic brain injuries. Further studies are required to explore how esRAGE can prevent neuronal damage in brain ischemia. It is conceivable that endothelial RAGE is a double-edged sword in brain ischemia; intracellular RAGE signal transduction can induce vascular inflammation and result in the derangement of microvascular circulation, while RAGE can transport the neuroprotective esRAGE into the brain and prevent neuronal cell death. Thus, a condition enriched with esRAGE could be neuroprotective in ischemia.

In conclusion, we confirmed that esRAGE could prevent neuronal cell death in the BCCAO ischemic mouse model and could be transferred into the brain through the BBB, where it accumulates around the neurons and functions as a neuroprotective factor. Accordingly, we suggest that a supplementation of esRAGE in ischemic brains will be a potential therapeutic tool for the prevention of neuronal cell death.
